# The Role of Pet Companionship in Older Adults’ Mental Health: A Qualitative Study during the Covid-19 Pandemic

**DOI:** 10.1192/j.eurpsy.2024.336

**Published:** 2024-08-27

**Authors:** S. von Humboldt, S. Silva, I. Leal

**Affiliations:** ^1^William James Center for Research, ISPA – Instituto Universitário; ^2^ISPA – Instituto Universitário, Lisbon, Portugal

## Abstract

**Introduction:**

Human functioning in the physical, emotional, psychological, and social arenas is improved by contact with animals.

**Objectives:**

This study aimed to explore the affective relationship of older adults with their pets during the COVID-19 pandemic; and to assess how the affective relationship with pets contributed to older adults’ mental health.

**Methods:**

This qualitative study included 176 participants aged 65-88 years from Portugal, the United Kingdom, and Spain. All interviews went through content analysis.

**Results:**

Findings indicated five themes: (1) Meaningful Emotional Relations (86%); (2) Intimacy with Partners (68%); (3) New Emotional Connections (61%); (4) Sensory Stimulation (55%); and; (5) Physical Enjoyment (23%). As for the second objective, three main themes emerged from the content analysis: (1) Decreasing Depressive and Anxiety Symptoms (73%); (2) Easing Feelings of Loneliness (68%); and (3) Reducing Mood Swings (43%).

**Conclusions:**

The affective relationship between older adults and their pets was relevant during the COVID-19 pandemic, since it allowed for solidifying affection relationships with their partner and creating new ones. Additionally, it contributed for deepening the affective relationship with themselves, in terms of sensory stimulus, physical touch and physical pleasure. Owning a pet, was important for improvement in mental health symptoms, associated to depression, anxiety, and mood swings.

Keywords: Affective relationship; animal companionship; COVID-19 pandemic; mental health; older adults; pets.

**Disclosure of Interest:**

None Declared

